# Status and Prospect of Drilling Fluid Loss and Lost Circulation Control Technology in Fractured Formation

**DOI:** 10.3390/gels8050260

**Published:** 2022-04-21

**Authors:** Jingbin Yang, Jinsheng Sun, Yingrui Bai, Kaihe Lv, Guodong Zhang, Yuhong Li

**Affiliations:** 1 School of Petroleum Engineering, China University of Petroleum (East China), Qingdao 266580, China; yangjingbin2018@163.com (J.Y.); smart-byron@163.com (Y.B.); lvkaihe@upc.edu.cn (K.L.); zhangguodong1106@126.com (G.Z.); 2 CNPC Engineering Technology R&D Company Limited, Beijing 102206, China; 3 Xi’an Institute of Measurement and Testing Technology, Xi’an 710068, China; liyh2006@163.com

**Keywords:** fractured formation, drilling fluid, lost circulation control mechanism, lost circulation material, evaluation method

## Abstract

Lost circulation in fractured formation is the first major technical problem that restricts improvements in the quality and efficiency of oil and gas drilling engineering. Improving the success rate of one-time lost circulation control is an urgent demand to ensure “safe, efficient and economic” drilling in oilfields all over the world. In view of the current situation, where drilling fluid loss occurs and the plugging mechanism of fractured formation is not perfect, this paper systematically summarizes the drilling fluid loss mechanism and model of fractured formation. The mechanism and the main influencing factors to improve the formation’s pressure-bearing capacity, based on stress cage theory, fracture closure stress theory, fracture extension stress theory and chemical strengthening wellbore theory, are analyzed in detail. The properties and interaction mechanism of various types of lost circulation materials, such as bridging, high water loss, curable, liquid absorption and expansion and flexible gel, are introduced. The characteristics and distribution of drilling fluid loss in fractured formation are also clarified. Furthermore, it is proposed that lost circulation control technology for fractured formation should focus on the development of big data and intelligence, and adaptive and efficient intelligent lost circulation material should be continuously developed, which lays a theoretical foundation for improving the success rate of lost circulation control in fractured formation.

## 1. Introduction

Safe, economical and efficient drilling is the key premise to accelerating the process of deep and ultra-deep oil and gas exploration and development and to increasing oil and gas production [1]. However, deep and ultra-deep formation fractures are widely developed, and lost circulation frequently occurs in the drilling process. Thus, the success rate for one-time plugging and controlling lost circulation is low [2]. Existing domestic technology and the comprehensive introduction of foreign technology have not solved this, and it is the first major technical problem restricting the improvement in the quality and efficiency of oil and gas drilling engineering [3]. During the drilling process, lost circulation will lead to failure of the drilling rig, greatly increasing the non-productive time and resulting in significant economic losses [4]. According to statistics, the China National Petroleum Corporation (CNPC, Beijing, China), Sinopec, China National Offshore Oil Corporation (CNOOC, Beijing, China), Yanchang Petroleum, etc., experience direct economic losses of more than 10 billion yuan per year [1]. In recent years, the average annual loss time caused by lost circulation encountered by the China National Petroleum Corporation (CNPC) was over 4000 days, accounting for more than two-thirds of the total complex loss time of drilling accidents, with an annual direct economic loss of over 5 billion yuan [5]. Imperfections in the plugging material and drilling fluid technology is the fundamental cause of these economic losses, due to the lost circulation [6]. The development of a new plugging theory, new material and new technology is key to reducing the economic losses caused by lost circulation.

The establishment of an accurate and effective drilling fluid loss model is key to studying the drilling fluid loss mechanism in fractured formations [7,8]. According to the key data parameters of drilling fluid loss, the pressure plugging dynamic model of fractured formation can be established, and then a series of strengthening theories is formed to improve the pressure-bearing capacity of fractured formation, which provides a guaranteed selection of reasonable lost circulation materials and lost circulation control measures [9]. Lost circulation material is the basis for successful lost circulation control [10]. The effect of lost circulation material is determined by its applicability to loss conditions and types of drilling fluid [11]. Lost circulation characteristics determine the key performance parameters of lost circulation materials [12]. To clarify the characteristics and distribution of drilling fluid loss in fractured formation, the causes of drilling fluid loss in fractured formation need to be analyzed in depth, and the subsequent proposal of a new method to classify drilling fluid loss types is one way of preventing drilling fluid loss [13,14,15]. It is also an important guarantee of improvements in the pressure-bearing capacity of fractured formation. 

In view of the current situation, where lost circulation control technology has not effectively solved the challenge of drilling fluid losses, this paper systematically summarizes the drilling fluid loss mechanisms in fractured formation. The mechanism and main influencing factors for improvements in the formation’s pressure-bearing capacity using stress cage theory, fracture closure stress theory, fracture extension stress theory and chemical strengthening wellbore theory are analyzed in detail. The properties and interaction mechanism of various types of lost circulation materials, such as bridging, high water loss, curable, liquid absorption and expansion and flexible gel, are introduced. The characteristics and distribution of drilling fluid loss in fractured formation are defined, and the development trends in lost circulation control technology in fractured formations are presented, providing a theoretical foundation for the scientific and efficient realization of stable plugging in fractured formations.

## 2. Drilling Fluid Loss and Lost Circulation Control Mechanism in Fractured Formation

At present, malignant lost circulation in fractured formations has become a worldwide problem that restricts the progress of oil and gas exploration and development. Improving the success rate of the one-time plugging of malignant lost circulation is an urgent demand for ensuring “safe, efficient and economic” drilling in oilfields around the world. It is one of the key points in the field of drilling engineering research and practice for revealing the loss mechanism of drilling fluid in fractured formation, developing special lost circulation materials, and establishing a strong applicable lost circulation control technology.

### 2.1. Mechanism of Drilling Fluid Loss in Fractured Formation

Fracture formation can be divided into fracture loss, fracture expansion loss and differential pressure loss, according to different loss mechanisms [16]. Fracture formation loss occurs mainly in limestone, dolomite, igneous rock, fault, fracture zone and other formations during drilling, including natural fracture loss and induced fracture loss [1]. Research into the mechanism of drilling fluid loss in fractured formation has mainly focused on the relationship between drilling fluid loss and parameters such as pressure, time, fracture characteristics and the rheological properties of drilling fluid [17]. At present, scholars at home and abroad mainly establish mathematical models and statistical models of drilling fluid loss in fractured formation to realize the inversion of the distribution and scale of underground fractures and then study the mechanism of drilling fluid loss in fractured formations [8,18,19].

It was necessary to analyze the cause of loss based on regional structural characteristics and a formation loss pressure profile to establish the mechanism model of drilling fluid loss in fractured formation [20]. In addition, the loss layer is further divided into fine interpretation, the size of the loss channel is quantitatively analyzed, and a reasonable loss model is established according to the factors affecting the drilling fluid loss rate. According to non-Newtonian fluid mechanics and fracture deformation behavior, serious problems for continental strata fracture drilling-fluid loss in northeast Sichuan led to the establishment of the natural loss and fractures extensibility loss mathematical model [21]. The simulation results were compared with the natural fracture loss evaluation experiment and the actual loss in the oilfield, and the error was within the reasonable control range. Based on fractal theory, Xu et al. carried out a drilling fluid loss dynamics simulation of natural fractures [22]. The Herschel-Bulkley (H-B) model was used to describe the non-Newtonian rheological characteristics of drilling fluid and the nonlinear deformation characteristics of fractures, and a two-dimensional drilling fluid loss model of rough fractures was established [23]. The effects of mesh size, fractal dimension and standard deviation on drilling fluid loss rate and cumulative loss were analyzed, which provided theoretical help in the understanding of drilling fluid loss mechanisms and inverting fracture width.

According to the coupling effect of the fluid characteristics of drilling fluid migration in extended deformation fractures, a fluid–solid coupling model of fracture deformation and drilling fluid loss was established based on one-dimensional radial fracture and two-dimensional plane fracture loss flow mathematical model [24]. Based on the simulation results, combined with the loss parameters under a certain drilling pressure difference, the fracture width could be predicted, the dynamic process of drilling fluid loss and fracture deformation behavior could be revealed, and the loss behavior and characteristics of natural fractures and extended fractures could be explored. By considering the fluid pressure drop and fracture deformation in fractured formations, Dokhani et al. developed a mathematical model describing drilling fluid loss trends in fractured formations [25]. The validity of the model was verified using the published measurement data for drilling fluid loss in the Gulf of Mexico and automatic curve-fitting technology to match the model prediction with the measured data and determine the fracture width [26]. At present, the simulation of drilling fluid loss in fractures, conducted by domestic and foreign scholars, mostly focused on one-dimensional and two-dimensional smooth linear horizontal fractures, while simulations of non-Newtonian fluid loss in two-dimensional rough exponential fractures with a certain dip angle have been fewer. 

Dynamic fracture width prediction was of great significance to the loss control of ultra-deep fractured tight reservoirs. The connected fault boundary and nonlinear deformation behavior were seldom considered in fracture width prediction. Xu et al. determined the fracture deformation equation using fitting stress-sensitivity test data, and, based on the non-Newtonian fluid-loss dynamics theory, established a dynamic fracture width prediction model considering power law fluid, fracture exponential deformation equations and fault boundaries [27]. Through a parameter analysis, the influences of pressure difference, consistency coefficient, flow pattern index and fault boundary distance on dynamic fracture width were studied. A control strategy for drilling fluid loss in ultra-deep fractured reservoirs was proposed, and the prediction model for dynamic fracture width was established. Verified by oilfield data, this was successfully applied to an ultra-deep fractured tight reservoir in Tarim Basin. Based on the dual continuum model, Huang et al. established a mathematical model for the solute transportation of drilling and completion fluid in fractured formations, determined the distribution range of the loss damage zone, and quantitatively evaluated the loss damage degree of drilling and completion fluid [28].

In sum, it was crucial to establish an accurate and effective drilling fluid loss model in the study of drilling fluid-loss mechanisms in fractured formations. Based on the loss mechanism and law of drilling fluid in fractured formations, the loss dynamics model was established to accurately predict the deformation characteristics of fractured formation, and loss-type diagnosis and fracture-width inversion were carried out, which could provide a theoretical basis for the prevention and plugging of drilling fluid. If drilling fluid loss occurs during oilfield drilling, drilling parameters and drilling fluid parameters can be adjusted according to the established dynamic model of drilling fluid loss, or reasonable lost circulation materials and lost circulation control measures can be selected to minimize the influence of drilling fluid loss.

### 2.2. Lost Circulation Control Mechanism in Fractured Formation

To improve the pressure-bearing capacity of fractured formations and seal up the lost zone, experts and scholars at home and abroad have carried out a lot of research and analysis on the loss mechanisms in the drilling process. According to various parameters in the process of drilling fluid loss, the key data parameters, such as the properties of the loss layer, the loss pressure and the shape of the loss channel, were found, and the pressure-plugging dynamic model of fractured formations was established. A series of strengthening theories were formed to improve the pressure-bearing capacity of fractured formations. The representative ones oncluded stress cage theory, increasing fracture closure stress, increasing fracture extension stress, chemical strengthening wellbore theory, etc.

(1) Stress cage theory

Stress cage theory mainly uses solid particles to bridge and plug in the fracture This compresses the formation around the wellbore to produce additional annular stress near the wellbore zone, enhances the wellbore stress near the wellbore wall, increases the ability of the wellbore wall to bear borehole pressure, and prevents fracturing loss under high borehole pressure [29]. According to the stress cage theory, the shape and fracture opening of the bridge are related to the particle size of the bridge material, and the increase in stress is related to the pore pressure, the bridge size and the heterogeneity of in situ stress [30]. The realization of the stress cage effect is based on the high strength of the bridge plug supporting the stress cage formed by fractures, and the bridge plug is in a stable state during the plugging process. As stress cage theory improves the pressure-bearing capacity of the formation by supporting the fracture opening and then squeezing the surrounding formation, it requires the formation to have a high elastic modulus and low brittleness. Therefore, the formation types used by the stress cage theory are mainly the low-permeability-dense consolidation type, pore type and fracture-pore type.

(2) Fracture closure stress theory

Fracture closure stress is the force that maintains the closure of a fractured formation surface [31]. In the drilling process, if the formation wellbore pressure is less than the fracture closure stress, the fracture will be in the closed state and circulation losses will not occur. If the formation wellbore pressure is greater than the fracture closure stress, the closed fracture will reopen and loss will continue. Therefore, the basic principle of increasing fracture closure stress is to increase crack width. The fracture closure stress can be increased by squeezing the strata on both sides of the fracture. Most of the stress can be transferred to the shaft wall, so that the fracture re-opening pressure increases [21]. Compared with the stress cage method, the improved fracture closure stress method has lower requirements regarding the type, size and strength of filling materials, and the traditional lost-circulation materials, cement and adhesive systems, are all suitable for this method. Therefore, two conditions must be met for a successful plugging, improving the principle of fracture closure stress [32]. First, the lost circulation material must isolate the fracture tip from the wellbore pressure to prevent the fracture from continuing to extend. Second, fracture width must meet the closure stress to meet the drilling requirements.

(3) Fracture extension stress theory

Improving fracture extension stress means that, after solid particles in drilling fluid enter the formation fractures, they can fill and seal the fractures, isolate the fracture tip, block the fluid pressure in wellbore from transferring to the fracture tip, enhance the extension stress and stability of formation fractures, and effectively improve the formation-bearing capacity [33,34]. Two points should be considered in plugging fractured formations using fracture extension stress theory. The first is the distribution of solid particles. Only by selecting a reasonable distribution of solid particles can a dense plugging layer be formed in the drilling plugging process. This effectively isolates the fracture tip, and thus improves the fracture extension stress [35]. The second is the concentration of solid particles. A higher concentration of solid particles can improve the plugging effect of the fracture tip, and better prevent pressure from spreading to the fracture tip. Increasing fracture extension stress is a kind of strengthening method, along with drilling, which is suitable for plugging water-based or oil-based drilling fluids [36]. This can realize the strengthening effect of borehole walls without affecting normal drilling operations. Compared with the stress cage theory and the fracture closure stress, the fracture extension stress theory can increase the fracture extension stress by isolating the fracture tip without changing the stress state near the borehole wall, to improve the pressure-bearing capacity of the formation. This is easily formed in the fracture tip of high-permeability formations, but not easy to form in the fracture tip of low-permeability formations. The particle size of the plugging material used to control the fracture tip extension should have a wide distribution range. The limitation of fracture extension stress theory lies in the compatibility of fracture and solid particles and the selection of solid particles with a proper concentration.

(4) Chemical strengthening wellbore theory

The chemical wellbore strengthening method can not only control the salinity differences between drilling fluid and formation pore fluid, it can also strengthen the wellbore effect [37]. In addition, the physical and chemical reactions between lost circulation materials can be used to construct the sealing partition wall to realize the wellbore strengthening and the sealing of the lost zone. Li et al. introduced the fully coupled thermochemical-pore elasticity theory into the physical model of wellbore reinforcement and established a multi-field, coupled wellbore strengthening model [38]. By observing the variation in circumferential stress and fracture aperture, the influence of thermochemical effects on enhanced wellbore plugging was studied. It was found that anisotropic Young’s modulus and Poisson’s ratio were sensitive to circumferential stress, and solute diffusion coefficient and permeability anisotropy have little effect on wellbore strengthening in a low-permeability formation. Compared with short fractures, long fractures were more conducive to wellbore strengthening, and the bridge location was close to the fracture mouth [39]. The chemical strengthening wellbore theory requires that the material can fully adapt to and utilize the formation environment, such as the temperature, pressure and fluid [40]. In addition, the material remains stable during wellbore flow and does not bond or solidify. After injection into the formation, physical and chemical reactions such as self-cementation rapidly occur to form high-strength structures and maintain stability for a period of time. The limitation of chemical strengthening wellbore theory lies in whether a high-strength plugging layer can be formed after the plugging material enters the formation.

## 3. Lost Circulation Material and Performance of Fractured Formation

Lost circulation materials are the basis of, and key to achieving, successful plugging. Experts and scholars at home and abroad have developed a variety of lost circulation materials, such as bridging, high water loss, curable, liquid absorption and expansion, flexible gel, etc., and explored the properties of different types of lost circulation materials and their interaction mechanism with fractures.

### 3.1. Bridge Lost Circulation Materials and Properties

Bridge plugging is an the effective means of solving the problem of lost circulation in fractured formation. The plugging layer with a dense structure and high bearing capacity is formed by bridging, stacking and filling the fractures with different shapes and sizes of bridging lost circulation materials [41]. The bridge lost circulation material is composed of a granular, fibrous, sheet inert material according to a certain mass ratio and the particle size of the composite lost circulation material [42]. Commonly used bridging materials include walnut shell, calcium carbonate, fiber, mica, etc. However, conventional lost circulation materials are not compatible with the formation and cannot form high-strength plugging layers. Due to the influence of gravity settlement, erosion in the fracture and other factors, this is difficult to place in large fractures with a large width and high longitudinal extension, especially in karst caves, which results in a low pressure-bearing capacity for the plugging layer. Obviously, the type, geometry and mechanical properties of the bridging lost circulation material play a vital role in the bearing capacity of the plugging layer [43].

Drilling fluid loss easily occurs in the drilling process of deep and ultra-deep fractured formations. The fracture plugging layer formed by the bridge lost circulation material is unstable and damaged in a complex environment, such as a high-temperature, high-pressure and high-in-situ-stress environment, leading to the failure of lost circulation control [11]. Kang et al. established an evaluation method and index system for the high-temperature aging performance of bridging lost circulation materials in deep and ultra-deep wells by analyzing the morphology, particle size distribution and mechanical properties of bridging lost circulation materials such as walnut shell and calcium carbonate [44]. The hermite interpolation method can ensure that the function value and the derivative value of the interpolated function at the constructed interpolation polynomial node are equal, and the piecewise low-order Hermite interpolation method can avoid the runge phenomenon caused by the increase in interpolation nodes, and meet the requirements of numerical solution accuracy for general engineering problems. Based on the measured particle size distribution data for single bridging lost circulation material, Zhu et al. proposed a new method to predict the particle size distribution of bridging lost circulation material and formula using the piecewise cubic Hermite interpolation method [45]. To solve the problems of poor retention capacity and the low plugging success rate of conventional bridging lost circulation materials, Li et al. determined the particle size range and volume ratio of different bridging materials, and formed a high-temperature and high-strength cross-linking plugging formula [46].

### 3.2. High-Water-Loss Lost Circulation Materials and Properties

High-water-loss lost circulation material refers to the lost circulation material that forms a dense plugging layer through solid-phase water loss and by filling in the loss channel [11]. High-water-loss lost circulation materials are generally composed of polymer, diatomite, cement, sepiolite, attapulgite, asbestos powder, stone ridge, filtration materials and inert materials in a certain proportion [19,47]. This kind of lost circulation material will rapidly lose water under the difference between the formation pressure and the drilling fluid column pressure, and the solid phase components will coagulate and thicken to quickly form a film or filter cake to seal the loss channel of the fractures. High-water-loss lost circulation materials are easy to use, and have a quick effect and high success rate in high-permeability strata [48]. However, the high-water-loss lost circulation material is similar to the bridge lost circulation material, which has poor adaptability to the loss channel and is easily washed away in larger fractures or caves [5]. Therefore, high-water-loss lost circulation material is can solve the vertical and horizontal loss zone and the formation of small loss.

Common high-water-loss lost circulation materials are Chevron’s Diaseal M lost circulation material, China’s DSL plugging agent and Z-DTR plugging agent [1]. The lost circulation material with high water loss, high pressure and high acid solubility, mainly composed of fine fiber and granular components, can quickly accumulate lost circulation material and block the loss channel. In the process of waiting plugging, the lost circulation material produces a cross-linking chemical reaction, and finally forms the plugging wall with high-pressure capacity [49]. By optimizing the plugging process and using high-water-loss lost circulation material, the successful one-time lost circulation control with multiple loss points in the same open hole was realized in the Jianmen 1 well, and the plugging effect was obvious. 

### 3.3. Curable Lost Circulation Materials and Properties

Curable lost circulation material refers to lost circulation material with strong thixotropic and curing properties, which can be quickly cured and blocked in the loss layer [1]. Curable lost circulation materials are generally composed of a curing agent, suspension stabilizer and retarder and other materials. Curable lost circulation materials can effectively reside in the lost formation after entrance. Due to its strong thixotropic performance and flow resistance in the loss layer, curable lost circulation material can quickly solidify and coagulate to form a solidified body with high strength, as well as reducing mud and drilling fluid loss [50]. Curable lost circulation material has wide sources, low cost, high strength, a simple preparation process and a high cementation strength after curing. However, its construction safety risk is high, and its resistance to high-salinity formations and water pollution are poor, which can easily lead to problems such as stuck drilling or its being diluted and washed away in fractures or karst caves [51].

Sentinel CemTM cement, developed by the Halliburton company, the MAGNE-SET type curable lost circulation material developed by Baker Hughes Company, HDL-4, and chemical consolidation and cross-linking film composite lost circulation material are common existing curable lost circulation materials [1]. However, in general, curable lost circulation materials have a higher bearing capacity and better curing performance than conventional bridging lost circulation materials. In the process of circulating drilling, the plugging layer will not be destroyed by the pressure of a drilling fluid column, which can greatly reduce the number of plugging operations. When the curable lost circulation material is used with an inert material, the lost circulation control success rate can be effectively improved. Inert materials can bridge and fill the lost layer to achieve the effect of “sealing the door”, while curable lost circulation materials can effectively stay in the lost layer after entering the lost layer to inhibit the loss of drilling fluid [52]. In addition, the curing agent can quickly solidify and condense to form a high-strength plugging layer and improve the lost circulation control effect. When curable lost circulation material is used with a cross-linked polymer, it can form a more stable three-dimensional network structure and achieve the best lost circulation control effect.

### 3.4. Liquid Absorption Expansion Lost Circulation Materials and Properties

Most of the liquid absorption expansion lost circulation materials are composed of water absorption and oil absorption resin materials, either alone or combined with other lost circulation materials [53]. Its molecular structure contains hydrophilic or lipophilic groups. It swells in water or oil, but does not dissolve. Absorbent expansion polymer lost circulation materials can be generally divided into anionic, cationic, non-ionic and composite ionic. The swelling polymer lost circulation material has the advantages of expansion and deformation and is not affected by the shape and size of the loss channel. It can solve the adaptive plugging problem that the bridge and high-water loss lost circulation material cannot solve [2]. Liquid absorption expansion lost circulation materials, using intermolecular van der Waals force and osmotic pressure difference for the fracture, can absorb water (oil) volume expansion, and form a good elastic filling layer. They are suitable for porous and fractured formations. However, their suction delay effect and temperature-resistance performance are poor, and their plugging applicability is poor in formations with large fractures or karst caves.

Common liquid absorption expansion lost circulation materials are acrylamide–acrylonitrile copolymer, acrylic acid–acrylic acid sodium copolymer polymer swelling resin and its mixture, and other lost circulation materials. In view of the poor stability and low strength of water absorption resin, Wang et al. prepared a water absorption resin type plugging agent PQ with acrylic acid and acrylamide as experimental raw materials [54]. Water absorption rate increased more than 150 times, with good stability. At present, it has problems of poor environmental protection, low strength and high cost. Huang et al. developed a new type of water absorption expansion plugging agent by free radical polymerization using acrylic acid, acrylamide, bentonite, inert materials and initiator as raw materials, which can greatly reduce the cost [55]. Liu et al. developed a micron-sized deformable globular gel, supplemented with optimized flak-like and fibrous lost circulation materials, and synthesized a globular gel compound plugging agent with oil absorption and expansion. Micro-holes and micro-fractures can be blocked while drilling to prevent or reduce oil-based drilling fluid loss.

### 3.5. Flexible Gel Lost Circulation Materials and Properties

Drilling in the process of formation environment becomes more demanding and the frequency of lost circulation is sharply increased with the gradual exploration and development of oil and gas under deep, ultra-deep, unconventional and other complex conditions [1]. Gel lost circulation materials mainly use chemical crosslinking reactions or interactions between molecules to form a high-strength gel with a three-dimensional network structure to plug the drilling fluid loss channels in complex formations [56,57]. Flexible gel is a kind of lost circulation material, which is suitable for different loss channels, mainly divided into crosslinking gel type and non-crosslinking gel type. Compared with other types of lost circulation materials, flexible polymer gel lost circulation materials can adapt to different scales of loss channels without being limited by their morphology because of their good deformability under compression, and easy to form high strength plugging in the loss channels [57].

Crosslinking gel refers to the injection of a polymer (or monomer), crosslinking agent and initiator into the downhole loss channel in the form of an aqueous solution [58]. The crosslinking reaction generates viscoelastic gel in the formation environment, and then blocks the loss channel [59]. Common cross-linked gels include cross-linked polymer gel, polyacrylamide cross-linked gel, composite nanoscale organic/inorganic gel, underground cross-linked polymer gel, etc. [60]. Non-crosslinked gels are mainly formed by an entanglement or association between polymer chains with special functional groups. In order to solve the problem of drilling fluid loss in geological drilling, Jia et al. prepared an environmentally friendly and strength-enhanced nano-silica composite gel for the temporary plugging of high-temperature reservoirs [61]. The gel had good mechanical and elastic properties. The addition of nano-silica made the three-dimensional network structure of the gel closer, which was conducive to enhancing the lost circulation control effect. Flexible gel lost circulation materials generally have poor high-temperature resistance and long-term stability under high-temperature conditions, resulting in ineffective fracture plugging or a high risk of re-leakage after plugging.

## 4. Evaluation Method of Drilling Fluid Loss and Lost Circulation Control

Evaluation method of drilling fluid lost circulation control is the key to preventing the loss of drilling fluid and an important basis for the choice of plugging measure. It helps to clarify the characteristics and distribution law of the loss of drilling fluid and establish the dynamic model of the loss of drilling fluid.

### 4.1. Evaluation Method of Drilling Fluid Loss in Fractured Formation

The determination of the lost zone is the key to successful lost circulation control during drilling. The accurate determination of lost circulation location within a short time can effectively improve the success rate of lost circulation control. There are many kinds of classification methods for drilling fluid loss in fractured formation. The classification of drilling fluid loss in fractured formation can reflect the mechanism of drilling fluid loss from different sides. Among them, the most commonly used method of drilling fluid loss classification is based on the characteristics of drilling fluid loss rate and drilling fluid loss channel in fractured formation [62]. The classification method based on loss rate can reflect the severity of drilling fluid loss as a whole. The classification method based on the characteristics of loss channels reflects the basic form of loss space in fractured strata to a certain extent. At present, foreign countries mainly use well-temperature testers, acoustic testers, eddy current testers, radioactive tracers and other instrument testing methods to determine the location of lost circulation points [8,15]. Domestic experts and scholars have also carried out some active and effective methods to find the lost circulation, such as well-temperature logging, fuzzy identification of the loss zone before the drilling risk prediction method, the comprehensive analysis method, etc. [63,64]. To solve the problem of drilling fluid loss in fractured formation, Zhang et al. divided drilling fluid loss in fractured formation into fracturing loss, expansion loss and differential pressure loss [65]. In order to clearly understand the loss behavior of drilling fluid in rough fractures and fracture networks, experts and scholars at home and abroad established one-dimensional linear, two-dimensional plane fracture and three-dimensional fracture network loss dynamics models based on fractal and Monte Carlo theories, and systematically analyzed the loss law for drilling fluid in fractured formations [66,67]. To understand lost circulation and optimize lost circulation control technology, Xu et al. conducted a study on the diagnosis of drilling fluid loss, established a two-dimensional plane fracture H-B flow model, and revealed the dynamic behavior of drilling fluid loss and its influencing factors [68].

To truly reflect the stress state of fractured formation and the law of fluid loss and flow, Sui et al. invented an experimental device and evaluation method to simulate the dynamic loss and plugging of drilling fluid [69]. This can simulate the positive and negative circulation dynamic loss process of drilling fluid on different core faces under original in situ stress conditions. To accurately grasp the loss situation, and adopt a reasonable and effective lost circulation control method to reduce the loss of drilling fluid, Bai et al. invented a method for predicting drilling fluid loss in fractured formations [70]. The loss of drilling fluid in fractured formations could be accurately predicted by calculating the fracture width, measuring the viscosity coefficient, flow characteristic index and yield stress of drilling fluids. To accurately measure and monitor the status of drilling fluid loss points in fractured formations and evaluate the loss strength of drilling fluid loss points, Jiang et al. invented a drilling fluid loss monitoring system and monitoring method based on the distributed optical fiber sensor [71]. This method could be used to design a lost circulation control technology scheme and conducts measurements according to the loss strength to protect drilling holes and drilling rigs.

### 4.2. Evaluation Method of Pressure-Bearing Capacity in Fractured Formation

The pressure-bearing capacity of fractured formations is an important index to measure the loss control effect of drilling fluid. It is of great significance for the efficient development of oil and gas resources in fractured formations to effectively improve the formations’ pressure-bearing capacity, establish the pressure-plugging prediction model of fractured formations and form the pressure-bearing-capacity strengthening method. At present, the performance of gel lost circulation materials is mainly evaluated by devices and methods such as high-temperature and high-pressure water loss meters, artificial core-simulation crack loss plugging, nano-composite organic/inorganic gel plugging agent devices, and high-temperature and high-pressure water loss meter sand bed evaluation experiments [72,73]. To solve the problem of lost control when drilling fluids in deep fractured formations, Xu et al. constructed an instability model for the plugging layer in fractured formation based on particle mechanics [74]. Based on the strength model of the fracture plugging layer, the instability mechanism of the fracture plugging layer was revealed. In addition, considering the stratum’s high-temperature, high-pressure and high-stress conditions, a new type of lost circulation material was selected to establish a quantitative scoring model for lost circulation materials in fractured formations, and a quantitative scoring optimization method was created for lost circulation materials in fractured formations.

An artificial fracture is used to simulate the loss formation channel in the commonly used lost circulation control evaluation devices. Zhu et al. provided a kind of fracture lost circulation control instrument, which could solve the problem with the existing lost circulation control evaluation device, which finds it difficult to simulate the formation loss channel [45]. Experts and scholars at home and abroad generally use the American Petroleum Institute (API, The United States) lost circulation control evaluation experimental device to evaluate and study the plugging ability of lost circulation materials. However, American Petroleum Institute (API, The United States) cannot simulate the re-opening loss of fractures caused by fracture closures under pressure. Therefore, She et al. invented a drilling-fluid pressure plugging fracturing test device [6]. This method can simulate the formation process of real fractures under the action of drilling fluid and the pressure plugging process of lost materials, which provides a new testing method for the research and evaluation of pressure plugging of drilling fluid under the condition of easy to lose formations. The innovation and breakthrough of the basic research on efficient lost circulation control materials and their action mechanisms are key to improving the success rate of lost circulation control in fractured formations.

The following technical challenges occur in the study of lost circulation control evaluation methods and theories in fractured formations: (1) The loss and plugging mechanisms of drilling fluid in fractured formations are not clear; therefore, scientific predictions of the actual properties of lost circulation formation (location of lost circulation, width of fracture and lost circulation pressure) cannot be supported, and technological research, as well as the development of lost circulation control materials, cannot be scientifically guided. (2) The existing bridge plugging materials poorly match the characteristics and types of lost circulation and lack a quantitative evaluation and lack optimization methods of bridge plugging materials for fractured formation. Additionally, the formation and pressure evolution mechanisms of the bridge plugging layer are unclear, and there is no scientific basis for bridge plugging formula design, resulting in a low success rate.

## 5. Development Prospect of Lost Circulation Control Technology 

### 5.1. Strengthening the Mechanism of Lost Circulation Control in Fractured Formation

At present, great progress has been made in the research aiming to improve formation-bearing capacity, but the lost circulation control mechanisms of fractured formation are still not perfect. Most theories only include a qualitative cognition, rather than quantitative calculation, and several theoretical models are independent of each other, leading to shortcomings. At the same time, the existing research is mainly focused on the lost circulation control mechanism of rigid granular materials, and there are few studies on the lost circulation control mechanism of flexible materials. In particular, the migration, filling and plugging mechanisms of flexible or composite lost circulation control materials in three-dimensional fractures needs further research. Therefore, the lost circulation control mechanisms have great limitations in their ability to guide the research and development of lost circulation control technology and field construction. Additionally, the site construction of a malignant lost circulation has high levels of blindness and the lost circulation control effect is not ideal.

In view of the above problems, more attention should be paid to studying the mechanisms of lost circulation control in fractured formations. The lost circulation control mechanism of drilling fluid loss was accurately revealed regarding the loss channel, mechanical balance and lost circulation control methods, and the laws of drilling fluid loss in different formations and at different scales were clarified. The migration, filling and plugging mechanisms of flexible or composite lost circulation control materials in three-dimensional fractures should be studied. This would provide strong basis for the scientific and efficient selection of lost circulation control materials, formulae, methods and technologies.

### 5.2. Research and Development of Adaptive and Efficient Lost Circulation Materials

In recent years, many kinds of lost circulation control materials have been developed by experts and scholars at home and abroad, and all of them have achieved good results to a certain extent. However, the available lost circulation control materials are limited for seriously fractured formations. For the fractured-vuggy formation, which is prone to malignant lost circulation, it is difficult for the lost circulation control material to form a high-strength plugging layer. At present, most of the bridging, gel and composite lost circulation control materials are used to effectively solve the problem of lost circulation in fractured formations. The success rate of lost circulation control for malignant lost circulation in fractured formations is low. Therefore, with the rapid development of material science theory and technology, it is important to promote the development of an intelligent lost circulation control theory for drilling fluid with an efficient lost circulation control material.

The compatibility between conventional lost circulation control material and complex fractured formation is poor, so the grading relationship between lost circulation control materials and complex fractured formations should be determined according to drilling fluid loss and plugging mechanism, to avoid the “sealing door” phenomenon. Attention should be paid to the research and development of adaptive lost circulation control materials with fracture space morphology. Conventional lost circulation materials have a weak residence ability in large-scale fracture space, it is difficult to effectively fill the space, and the plugging effect is not good. According to the parameters of the fracture loss channel and the physical and chemical characteristics of the material, an efficient lost circulation control material with a strong residence and full filling effect should be developed. By clarifying its self-adaptive filling law in the loss channel, the bearing capacity can be effectively improved. 

In view of conventional lost circulation control materials’ lack of high-temperature resistance in deep and ultra-deep high temperature wells, it is difficult to achieve long-term stable plugging. Therefore, we should pay attention to the research into the high-temperature resistance of lost circulation control materials. The research and development of high-temperature lost circulation control materials for deep and ultra-deep high-temperature wells could improve the success rate of the lost circulation control.

### 5.3. Intelligent Plugging Equipment and Technology

At present, lost circulation control technology of fractured formations at home and abroad is mainly based on the plugging treatment experience of similar well conditions or neighboring loss wells. There is no scientific optimization and evaluation expert system for lost circulation control technology, and no unified lost circulation control technology specification has been formed. American Petroleum Institute (API) lost circulation control evaluation experimental devices are used to evaluate and study the pressure-bearing capacity at home and abroad. However, the lost circulation control devices cannot simulate the re-opening of fractures after they are closed under pressure. Therefore, with the expansion and application of the big data era and artificial intelligence technology in the field of oil and gas exploration and development, intelligent lost circulation control equipment and technology will become an inevitable development trend.

In view of the existing lost circulation control technology, which has strong experience and low intelligence, it is difficult to effectively solve the problems of drilling fluid loss in deep and ultra-deep fractured formations. Therefore, we should pay attention to the development of big data and intelligent lost circulation control technology. Firstly, the data for key areas of drilling fluid loss should be summarized into a database to form a universal expert system for lost circulation control prediction and analysis. Secondly, the research and development regarding intelligent lost circulation control materials and key equipment should be strengthened, promoting the rapid development of lost circulation control methods and technologies in the intelligent direction, and realizing the efficient and intelligent lost circulation control technology for deep and ultra-deep fractured formations.

## 6. Conclusions

(1) Lost circulation, especially in fractured formations, is the first major technical problem restricting improvements in oil and gas drilling quality and efficiency. The mechanism of drilling fluid loss in fractured formations is revealed, a dynamic model of drilling fluid loss in fractured formations is established, and a strengthening theory to improve the pressure-bearing capacity of fractured formations is formed, which can provide a theoretical basis for the lost circulation control of drilling fluids.

(2) Lost circulation materials form the basis of, and key to, successful lost circulation control. Experts and scholars at home and abroad have successively developed various types of lost circulation materials such as bridging, high water loss, curable, liquid absorption expansion and flexible gel, which can solve the problem of lost circulation in fractured formations to a certain extent. However, efficient lost circulation materials that can effectively solve the loss of drilling fluid in deep and ultra-deep high-temperature wells are lacking, and it is important to develop efficient intelligent lost circulation materials to achieve the long-term and stable plugging of fracture formations.

(3) The pressure-bearing capacity of fractured formations is an important index to measure the loss control effect of drilling fluids. This is key to improving the lost circulation control success rate of fractured formations, clarifying the characteristics and distribution law of drilling fluid loss, deeply analyzing the causes of drilling fluid loss, and forming a method to strengthen the pressure-bearing capacity of fractured formations.

## Data Availability

All persons included have agreed to confirm.
